# Distinct attitudes of professionals from different medical specialties toward autonomy and legal instruments in the assessment of patients with Alzheimer’s disease

**DOI:** 10.1590/S1980-57642010DN40200005

**Published:** 2010

**Authors:** Ana Beatriz Maringolo Pioltini, Cristiane Lara Mendes-Chiloff, Arthur Oscar Schelp, Everson da Silva Marcolino

**Affiliations:** 1 Fourth-year Medical Student, Botucatu Medical School, São Paulo State University (UNESP), Botucatu SP, Brazil. FAPESP Fellows.; 2 Psychologist, Department of Neurology, Psychology, and Psychiatry, Botucatu Medical School, São Paulo State University (UNESP), Botucatu SP, Brazil.; 3 Neurologist, Department of Neurology, Psychology, and Psychiatry, Botucatu Medical School, São Paulo State University (UNESP), Botucatu SP, Brazil.; 4 Lawyer, São Paulo Federal Justice Department.

**Keywords:** Alzheimer’s disease, attitudes, ethical and legal aspects

## Abstract

The evaluation of competence of Alzheimer’s disease (AD) patients to assume personal or collective responsibilities and the resulting legal implications is a relevant issue.

**Objectives:**

The aim of this study was to evaluate the attitudes of different medical specialists towards the disability of patients with Alzheimer’s disease and practitioners’ competence to interfere with decision-making autonomy.

**Methods:**

Professionals from different areas (Neurology, Psychiatry, Geriatrics, and General Practice) were interviewed by one of the authors, after being presented a fictitious clinical case which raised several topics, namely:

[1] Critical judgment and capacity of the patient to take decisions related to daily activities;
[2] The role of family physicians in nominating trustees and caregivers.

**Results:**

Answers to the first question did not differ regarding degree of preservation of awareness but at least 25% stressed that the patient must be carefully listened to, independent of caregiver or legal representative opinion. There were significant knowledge gaps in responses to the second question. Half of the physicians interviewed did not have adequate information about the legal aspects of caring for patients with Alzheimer’s disease.

**Conclusions:**

Legal aspects is a topic that must be incorporated into professional training in order to improve attitudes toward the long-term management of patients with dementia.

## Abstract (pt)

A avaliação da competência de pacientes com doença de Alzheimer (DA) para assumir responsabilidades pessoais ou coletivas e as implicações legais resultantes constituem um tema relevante.

**Objetivos:**

O objetivo do estudo foi analisar as diferentes atitudes de profissionais de diferentes áreas de formação frente à incapacidade de pacientes com doença de Alzheimer e sua competência para interferir com autonomia para tomada de decisões.

**Métodos:**

Profissionais das diferentes áreas de atuação (Neurologia, Psiquiatria e Geriatria e Medicina geral) foram entrevistados por um dos autores, depois de serem apresentados a um caso clínico fictício, no qual alguns tópicos foram levantados:

[1] Juízo crítico e capacidade do paciente para tomar decisões relacionadas ao seu cotidiano;
[2] Papel do médico na escolha e nomeação de cuidadores e tutores.

**Resultados:**

A resposta a primeira questão não evidenciou grandes divergências quando ao grau de incapacidade, mas 25% dos entrevistados chamaram atenção para o fato de que os pacientes devem ser ouvidos e avaliados, independentemente da opinião externada pelos cuidadores e representantes legais. Na resposta ao segundo quesito ficou evidente a falta de informações sobre aspectos legais e éticos relacionados aos tutores e cuidadores.

**Conclusões:**

Aspectos legais constituem um tópico que deve ser incorporado ao treinamento profissional para aprimorar condutas no tratamento de longo prazo de pacientes com demência.

The increasing life expectation in many countries around the world has led to growing concerns over implications of the competence of Alzheimer’s disease (AD) patients’ to assume personal or collective responsibilities and the resulting legal implications where this also includes reports of suicide among patients with Alzheimer’s disease.^1,2^

The decision spectrum includes aspects from daily life, such as driving^3^ and more complex issues such as: respecting their autonomy in deciding the destination and use of property, belongings, and other issues.^4^ The mental capacity of dementia patients and their ability to manage their interests hinges on the relationship between the competence of the individual and the society to which they belong.^5^ Determination of self-awareness, perceived knowledge, and perceived skills are measured and rated not only by the attending physician but also by a designated trustee. This matter is regulated by specific laws with distinct characteristics for each country. ^6,7^

Discrepancies among professionals’ competence regarding disability issues was reported by Strike et al.^8^ in a study that stratified experts into two groups; groups with more, or less, disability-related experience. The criteria were time (in years) of general experience, and the participants included psychiatrists, psychologists, social workers, career counselors, disability specialists, and other mental health professionals. The study showed a significantly higher score in the experienced group compared to the less experienced group on awareness, self-perception, and skills capacity.

In many cases a specific health professional is assigned. However, under the Brazilian public health system, no specific attending professional is nominated and the dementia patient could be referred to professionals of many different disciplines each with different ways of handling mental distress or specific training for handling physical skills. Thus, judgment of competency is unlikely to be the same for these different physicians. This is especially true in mild cognitive impairment, recently categorized as AD, when there is no designated legal trustee to act as guardian for the patient.

The aim of this study was to collect impressions from different specialists working in the area of dementia patient care concerning the disabilities affecting patients’ competence and autonomy to take part in all daily living activities, and to ascertain practitioners’ awareness of the role of caregivers and trustees.

## Methods

Data was collected between May and September 2009 on the perspective of health professionals from different specialist areas (Neurology, Psychiatry, Geriatrics, and General Practice) identifying similar or distinct concepts and perspectives about a fictitious clinical case. The questionnaire was presented to 20 individuals, 12 men and 8 women with between 1 and 45 years of medical practice in different medical specialties. Respondents were all blind to the opinions and conclusions of the other participants. Participation was individual to avoid the possibility of interaction between those taking part. The professionals were drawn from different specialties and were stratified as follows: 3 psychiatrists, 3 neurologists, 1 neurosurgeon, 4 pediatricians, 3 gynecologists, 4 geriatric specialists, and 2 pathologists.

### Fictitious clinical case

Relatives, including two married sons who live in another city, ask the doctor for a medical report on the patient’s disease and status. An unmarried daughter, who lives with the patient, is against the request and states that it is financially motivated.

#### The patient

A 68-year-old woman, widowed two years earlier, lives with her single 30-year-old daughter who works during the day and stays with her mother after lunch on Saturdays and Sundays. During weekday evenings she supervises and helps her mother with her personal hygiene. In the daughter’s absence, a 50-year-old lady living nearby is paid to help with hygiene, and provides meals, and other help needed. For the last three years, the patient has demonstrated difficulty in performing activities such as handling of the cash dispensing machine (ATM) and the remote control. She can no longer recall hours, days, and months, with constant errors in sequencing. After her husband’s death two years previous, she began to have difficulty distinguishing objects and substances, for instance between salt and sugar or identifying pairs of socks and shoes, including size (trying to wear her daughter’s shoes). She routinely visits neighbors, and for the last year the patient has not gone shopping in local supermarkets due to difficulties in managing money and mistakes in product choice. Complementary examination including computed tomography (CT) and magnetic resonance image (MRI) shows non-specific global atrophy without other findings.

The caregiver reports that the patient does not adequately perform personal hygiene and does not accept help. The patient receives a pension and rental income from two properties. She often asks questions about her earnings and is unable to remember whether they have already been collected. In recent months, she has presented forgetfulness related to sequence of events, characterized by the doctor as episodic memory impairment. Once a month, her daughter accompanies her to the bank to collect her income and pension.

Since her husband’s death, she consistently inquires whether Alzheimer’s disease affects her as it had affected one of her sisters who died 5 years earlier.

The questions presented by the medicine student relate to a hypothetical clinical case and basic issues pertinent to patients with AD. The questions were as follows:

[1] Views on critical judgment and the patient’s ability to make decisions related to their daily life and administration of personal and shared property;
[2] What should be the role of the physician in appointing caregivers and an attorney-in-fact?

All interviewees received a full explanation about the study and gave their informed consent. The Medical Ethics Research Committee of Botucatu Medical School - São Paulo State University, approved the study.

## Results

### Question 1. Views on critical judgment and the patient’s ability to make decisions related to their daily life and administration of personal and shared property.

- Two psychiatrists (one woman with less than 10 years’ medical practice, and one man with more than 10 years’ medical practice), two neurologists (both men with more than 10 years’ experience), and one pediatrician (woman with more than 10 years’ experience), who make up 25% of those interviewed, believed that the patient required evaluation in a medical consultation to confirm the daughter’s and carer’s reports.

#### Quotes

“Firstly I see the need to evaluate the patient in a medical consultation to evaluate if the report is true”

“As economic interests are involved, a more careful evaluation is required”

- Three pediatricians (all women with more than 10 years’ medical practice), one neurosurgeon (man with more than 10 years’ medical practice), four geriatricians (all men with more than 10 years’ medical practice), one gynecologist (woman with less than 10 years’ medical practice), one neurologist (man with more than 10 years medical practice), one psychiatrist (man with less than 10 years’ medical practice), and one pathologist (man with more than 10 years’ medical practice), who make up 60% of those interviewed believed that the patient was incapable of making decisions and required full support.

#### Quotes

“This patient is incapable of making decisions and carrying out daily activities and requires someone to help her, preferably a family member”

- Approximately 80% of this group (four geriatricians, one neurosurgeon, one pediatrician, one gynecologist, and one pathologist) supported the idea that the patient must be stimulated to perform her activities, albeit with help.

“The patient must keep her autonomy, but without exceeding her limits, which could generate a picture of stress”

- Twenty percent of this group (2 pediatricians with more than 10 years’ medical practice) believed this patient needed multiprofessional help: occupational therapy, nursing, and a restructuring of her environment. Other resources could also be employed: painting, handicrafts, etc.

#### Quotes

“The patient needs to be stimulated in activities that she gets pleasure from: painting, drawing, working in clay”

- One pathologist (woman with more than 10 years, medical practice) and two gynecologists (both men, one with less and one with more, than 10 years’ medical practice), who make up 15% of those interviewed felt unable to give their opinion on this case for lack of knowledge on the subject.

### Question 2. What should be the role of physicians regarding appointing caregivers and an attorney-in-fact.

- Practically 90% of those interviewed did not know the difference between carer and attorney-in-fact. Only two geriatricians (both men with more than 10 years medical practice) knew the difference.

Those unaware of the difference were given an explanation of the difference between carer and attorney-in-fact and the question posed again.

- One gynecologist (woman with less than 10 years’ medical practice), two psychiatrists (one woman and one man, both with less than 10 years’ medical practice), and three pediatricians (all women with more than 10 years’ medical practice), corresponding to 30% of those interviewed, believed that it falls to the doctor to request a place on the Medical Ethics Committee and recommend legal advice to define between caregiver and attorney-in-fact.
- Two male gynecologists (one with more and one with less than 10 years’ experience) and a female pathologist (with more than 10 years’ medical practice) corresponding to 15% of those interviewed believed that the doctor must advise the caregiver but not the attorney-in-fact.

#### Quotes

“The doctor does not have the right to interfere with the attorney-in-fact”

- One pediatrician (woman with more than 10 years’ medical practice), three neurologists, one neurosurgeon, one pathologist (woman with more than 10 years’ medical practice), four geriatricians, and one psychiatrist (man with more than 10 years’ medical practice), corresponding to 55% of those interviewed, considered that the physician needed to produce a pathology and prognosis report, and to suggest the role of the carer, with the legal system responsible for designating the attorney-in fact.

#### Quotes

“I believe that the role of the doctor is to report in a clear manner, based on clinical theory and after careful evaluation of the patient, the patient’s capacity for activities, reasoning, memory, and understanding”.

“It is up to the doctor to define the aim of integral patient care, based on clinical data and from the relationship/interaction with the patient”.

“The doctor must recommend legal advice and maintain follow-up, but not in designating the carer or attorney-in-fact”.

“They should suggest, with empathy, what is most suitable for improving their patient’s quality of life”.

## Discussion

The increasing prevalence of AD makes increased longevity, one of the great triumphs of modern society, a threat of epidemic proportions for which we are still not prepared.^9^ The aging process in the Brazilian population, which affects 7.1% of elderly Brazilians,^10^ is an increasing public health concern and a pattern which constitutes a long-term trend.^11^

In this study, physicians were presented with a hypothetical clinical case and questioned on basic needs and the family physician’s view on issues concerning legal aspects such as nominating caregivers and a legal representative (attorney-in-fact).

Most of those interviewed agreed that there is a major compromise in the patient’s cognitive abilities, at least with mild cognitive impairment. Some answers stressed the patient’s inability to make decisions and the need to receive help for all activities - *“This patient is incapable of making decisions and performing daily activities independently, she needs someone to help her, the relative being a good source of support”*, and *“The patient needs to maintain her autonomy, but without exceeding her own limits, which could generate a picture of stress”*. Analysis of responses revealed difficulties for the interviewees in discerning between autonomy and patient competence in performing activities and making decisions about their personal life. Importantly, 60% of those interviewed, independent of specialty and time in the profession stated that the patient was incapable of performing all the activities, making decisions or having autonomy. Defanti et al. called attention to the fact that autonomy is based on capacities that could be lost or disturbed with disease progression.^4^ A quarter of those interviewed expressed their concerns about interference from caregivers and relatives on establishing prognosis. The statement is translated by deponents as the requirement for diagnosis to arise from careful examination of the patient and then to be presented before relatives, caregivers and attorney-in-fact.

Diagnosis of a patient’s loss of awareness and mental deficits leads to a critical key point - the decision to certify the patient for civil rights. The second question exposed the inability of 90% of the participants to identify the difference between a caregiver and an attorney-in-fact. Health professionals need to have a clear understanding of the distinction that the caregiver is chosen by the family with help from health professionals. But the attorney-in-fact is chosen by a judge, upon request by any individual judged capable, with permission to make the request from the actual patient. The judgment may require medical and other evaluations. This possibility has to be explained to the family. In Brazil there is a law that can be assessed in the network at any time^7^ involving all steps, and which must be followed in order to start a process of nominating power of attorney.

A point to reflect on is that participants with 10 or more years in practice from areas less closely related to mental health believed that they did not have sufficient information to form an opinion. This highlights the need for additional provisions for all specialties. The hypothesis that some professionals are under more pressure to give opinions on a patient’s competence to assume responsibilities must be taken into account. Nevertheless, there are studies^12^ reporting only moderate agreement among experts in detecting impairment to decision-making abilities.

Other authors draw attention to the fact that despite knowing who the caregiver is, many physicians are not well informed on the role and forms of interaction with these professionals or relatives.^13^ Three of the specialists from areas not linked with mental health believed that the assisting doctor must advise the carer, but without informing the attorney-in-fact, i.e. the patient’s legal representative, on all aspects including decisions regarding autonomy. The need to designate a trustee reveals and highlights the importance of providing the patient with the best conditions of help and support, through greater autonomy and minimal dependence.

Contrasting opinion is apparent among different physician specializations, although time in practice represents an issue that can play a role in an area that calls for non-technical knowledge but requires legal information. With this in mind we cite a study where the diagnosis and therapeutic procedures of 94 general practitioners/interns and neurologists/psychiatrists, who dealt with psycho geriatric patients, were investigated by questionnaire.^14^ Data obtained suggested that there were no stark discrepancies between the different medical groups with regard to psycho geriatric care. However, the decision to request relatives to take actions which guarantee the patient complete respect of their expressed wishes up to or before the onset of the dementia picture, or during disease evolution is not related to diagnosis or therapeutic measures, but to an attitude toward maintaining the autonomy of the individual with dementia. It is evident that there was no homogeneous perception of the associated situational constraints related to the needs of the social group to which the patient belonged.

Another impression was a lack of knowledge on the national laws covering the competence to appoint power of attorney in cases of incompetence or functional incapacity. Efforts to intensify campaigns to provide physicians who take care of demented patients with information on juridical aspects represents a positive measure to assure broader care for patients and their families.

The results of this study highlight the need for more evidence-based decision-making regarding treatment, care, and guidelines. Emphasis on legal aspects is a topic that must be incorporated into professional training for assessing attitudes toward demented patients.
